# Pathological and Molecular Studies of *Neospora caninum* Infection in Aborted Bovine Foetuses in Khorasan Razavi Province, Iran

**DOI:** 10.1002/vms3.70329

**Published:** 2025-04-26

**Authors:** Asma Keyvanlou Shahrestanaki, Hossein Nourani, Gholamreza Razmi

**Affiliations:** ^1^ Department of Pathobiology Faculty of Veterinary Medicine Ferdowsi University of Mashhad Mashhad Iran

**Keywords:** abortion, cattle, histopathology, nested PCR, Neospora caninum, serology

## Abstract

**Background:**

*Neospora caninum* is an obligate intracellular protozoan that is well established as a causative agent of abortion in dairy cattle worldwide.

**Objectives:**

The objective of this study was to determine the role of *N. caninum* infection in the abortion in dairy cattle in the Khorasan Razavi Province, Iran.

**Methods:**

From 2022 to 2024, 105 aborted bovine foetuses were collected from dairy cattle in Khorasan Razavi province. Brain samples of aborted foetuses were tested using nested PCR and histopathological examination. In addition, blood samples were collected from dairy cattle that had aborted PCR‐positive foetuses and were analysed using enzyme‐linked immunosorbent assay (ELISA).

**Results and Conclusions:**

In the present study, *N. caninum* infection was detected in 24.76% (26 out of 105) of aborted bovine foetuses by nested PCR analysis. The brain tissues of 20 bovine‐aborted foetuses were only suitable for histopathological examination. Lesions of the central nervous system were severe hyperaemia, perivascular cuffing, astrogliosis, mild encephalitis and focal necrosis. One foetus exhibited a 32‐µm *N. caninum* cyst within the brain tissue. IgG antibodies against *N. caninum* were identified in all dairy cattle that aborted infected foetuses through ELISA testing. Molecular, histopathological and serological findings strongly suggest that *N. caninum* plays a significant role in bovine abortion in dairy cattle in Khorasan Razavi Province, northeast Iran.

## Graphical abstract

Aborted bovine foetuses were collected from dairy cattle in Khorasan Razavi province. Brain samples of aborted foetuses were tested to detect *N.caninum* infection using nested PCR and histopathological examination. In addition, blood samples were collected from dairy cattle that had aborted PCR‐positive foetuses and were analysed using ELISA

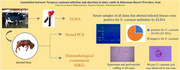

## 1 Introduction

*Neospora caninum* is a critical cause of abortion, stillbirth and congenital neosporosis in cattle (Abdelbaky et al. 2020; Idarraga‐Bedoya et al. 2020). This parasite belongs to the *Apicomplexa* phylum and was mistakenly diagnosed as *Toxoplasma gondii* before 1988 (Sykes et al. 2022). McAllister et al. (1998) described the parasite's life cycle. Different types of canids, such as domestic dogs (*Canis familiaris*), coyotes (*Canis latrans*), and grey wolves (*Canis lupus*), are definitive hosts of *N. caninum* and are infected by eating the tissue containing *N. caninum* cysts in the intermediate host (Zaghawa et al. 2023; de Souza et al. 2022). After the gametogony and sporogony stages in the small intestine of dogs, *N. caninum* oocysts are seen in faeces. The shedding period of the oocyst is short, and its number is small (Dubey et al. 2017). The size of non‐sporulated oocysts is approximately 10–14 µm, and 5 days after eating the tissue cysts, they are excreted in the faeces. Sporeling happens after 24–72 h outside the body (Sykes et al. 2022). Intermediate hosts, either through ingestion of oocysts with food and water (horizontal transmission) or through endogenous transmission (vertical transmission), are infected (Khan 2020; Lefkaditis et al. 2020). The size of tissue cysts varies according to the host species and infected cell type (Dubey et al. 2017). Rapid intracellular multiplication of tachyzoites leads to necrosis of the infected cells (Dubey et al. 2017). A widespread of tachyzoites to most organs of the host's body occurs in the acute phase of the disease, and following the host's immune response; the parasite enters the stage of slow reproduction and forms a cyst containing bradyzoites in the central nervous system or muscle tissue (Sykes et al. 2022). The rupture of tissue cysts is associated with granulomatous reactions in the affected tissue (Dubey et al. 2017).

Many studies have been conducted on the seroepidemiology of *N. caninum* in intermediate and final hosts in Iran and worldwide (Gharekhani et al. 2020). The prevalence of *N. caninum* infection in Iran is about −3.8% to 76.2% in cattle and 0.0%–54.6% in dogs. In molecular studies, *N. caninum* deoxyribonucleic acid (DNA) was detected in 11%–66.7% of aborted foetuses (Gharekhani et al. 2020). These studies have also shown that dairy cows are more sensitive to neosporosis than beef cows (Otranto et al. 2003). The probability of abortion in seropositive cows is 1.6 times higher than that in seronegative cows (Sykes et al. 2022).

This study aimed to determine the potential role of *N. caninum* in bovine abortion cases in dairy cows in Khorasan Razavi Province, northeast Iran. A multifaceted approach incorporating serological, histopathological and molecular techniques has been employed to achieve this aim.

## 2 Materials and Methods

### 2.1 Location

**FIGURE 1:**
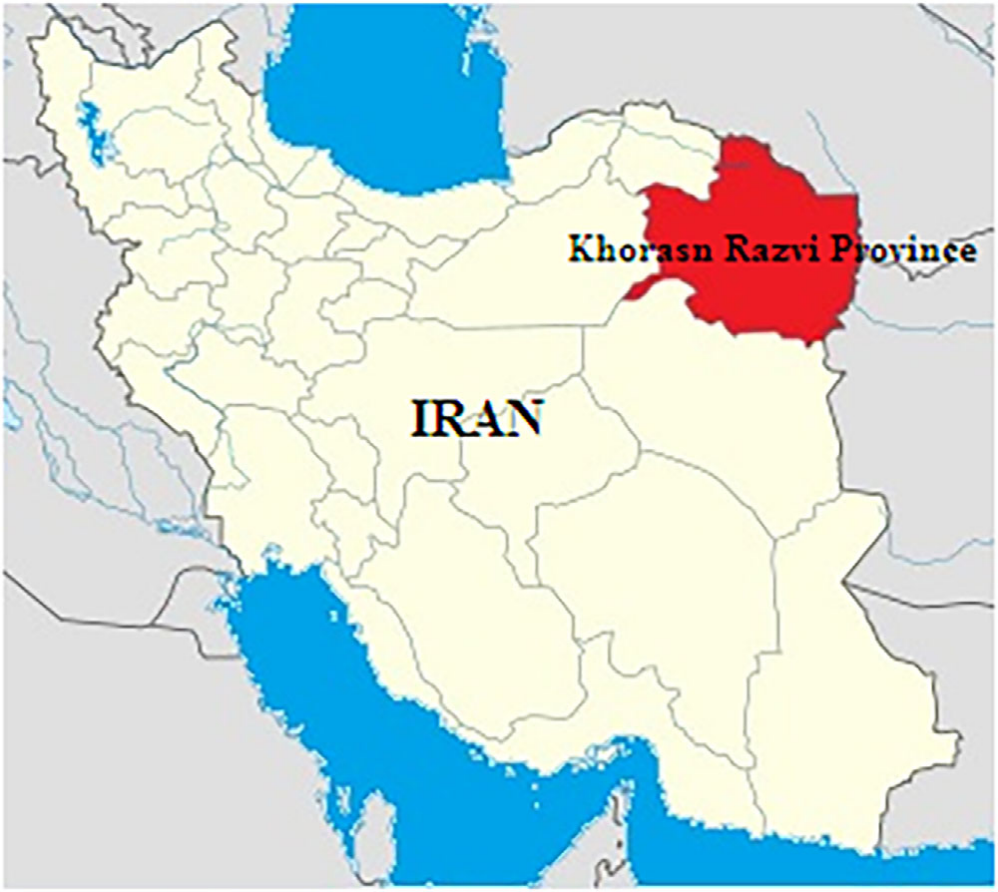
The location of Khorasan Razavi province in Iran.

The study was conducted in Khorasan Razavi province that located in northeastern of Iran (56°19′–61°16′ E and 33°52′–37°42′ N). The vast expanse of the province, along with factors such as the presence of high mountain ranges, desert areas, its distance from the sea and the influence of various winds, have resulted in diverse climates across different regions of Khorasan Razavi. The climate of Razavi Khorasan Province is predominantly arid and semi‐arid cold. Much of the province exhibits desert climate characteristics. Rainfall is predominantly concentrated in the cold season with significant variability, and the province experiences notable temperature extremes and varied humidity levels (Figure 1).

### 2.2 Sampling

One hundred and five samples were collected from submitted aborted bovine foetuses of dairy farms to the centre of excellence in ruminant abortion from 2022 to 2024 in the Khorasan Razavi province. First, the age of aborted foetuses were estimated by crown‐rump length and then the brain tissue was removed aseptically, with one part allocated for histopathology and another for PCR examination. All dairy cattle of these dairy farms were bred by artificial insemination and free of *Brucella* spp., bovine viral diarrhoea virus (BVDV), and infectious bovine rhinotracheitis virus (IBRV) based on serological and molecular examination.

The age distribution of 105 aborted dairy cows was documented as follows: 29 cows (28%) were 2 years old, 41 cows (39%) were 3 years old, 12 cows (11%) were 4 years old, and 23 cows (22%) were 5 years old.

### 2.3 DNA Extraction and PCR Amplification

Ten grams of each brain sample were collected in microtubes for DNA analysis. The tissue samples were stored at −20°C until DNA extraction. DNA was extracted from 20 mg of tissue using the Addverb Genomic DNA extraction kit (Better Bio‐Tech) according to the manufacturer's protocol. The DNA samples were stored at −20°C until the nested PCR test was performed.

The nested‐PCR test was performed on the internal transcribed spacer 1 (ITS1) region with primers NN1/NN2 and NP1/NP2 to trace the DNA of *N. caninum* by Buxton et al. (1998). The nested PCR protocol employed in this study precisely followed the conditions described by Buxton et al. (1998), including the exact temperatures and cycle numbers.

The nested PCR reaction was performed in a 25‐µL mixture containing 2 µL of total DNA, 10 µL of commercial premix master mix (Parstous co, Mashhad), 1 µL of each primer, and 11 µL of nuclease‐free water in a thermocycler. For nested PCR of the ITS1 region of *N. caninum*, the primer pairs NN1/NN2 and NP1/NP2 were used to amplify DNA fragments according to the procedure described by Buxton et al. (1998). The first amplification was as follows: 1 cycle of 95°C for 5 min, 26 cycles of 94°C for 1 min, 48°C for 1 min, 72°C for 1 min, and one process of 72°C for 5 min, and maintenance at 4°C. The second amplification was 26 cycles at 94°C for 1 min, 48°C for 30 s, 72°C for 30 s, and 1 cycle at 72°C for 5 min, using 2 µL of the first amplification product. DNA extracted from *N. caninum* tachyzoites and distilled water were used as positive and negative controls for PCR, respectively. The resulting products were subjected to electrophoresis on 2% agarose gel in TBE ×1 buffer and visualized using ultraviolet light. The size of the DNA fragments was compared with a standard molecular weight (100 bp DNA ladder). Samples were considered positive when a 249 bp band in size was present when NN1‐NN2/NP1‐NP2 primers were used.

### 2.4 Histopathology

Brain samples were taken aseptically and fixed in 25% buffered formaldehyde solution for histopathological analysis. The fixed tissues were processed using a tissue processor and embedded in paraffin wax. After the blocks were prepared, sections were serially cut into 5 µm using a microtome. Tissue sections were deparaffinized and stained with haematoxylin and eosin (H&E). The stained sections were histopathologically examined using light microscopy. This microscopic examination aimed to identify characteristic lesions suggestive of *N. caninum* infection.

### 2.5 Serology

Blood samples were collected from dairy cows that had aborted infected foetuses (PCR‐positive). These samples were subsequently analysed using a commercially available enzyme‐linked immunosorbent assay (ELISA) kit (ELISA kit; Bio‐X Diagnostics Co., Belgium) to detect the presence of antibodies against *N. caninum*. The diluent used in this kit was a specific buffer solution provided by the manufacturer, and the serum samples were diluted to a ratio of 1:100 using this diluent before being tested with the ELISA kit and analysed for the presence of IgG antibodies specific to *N. caninum*, as recommended by the manufacturer. A test sample is considered positive if its OD value is greater than the 15. Conversely, a sample is considered negative if its OD value is less than 10.

## 3 Results

**FIGURE 2:**
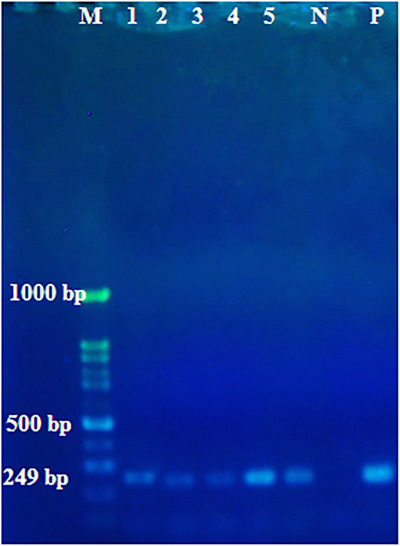
Electrophoresis results of nested PCR with special primers, M: marker, P: positive control, N: negative control, 1, 2, 3, 4 and 5: neospora positive samples (249 bp).

**FIGURE 3:**
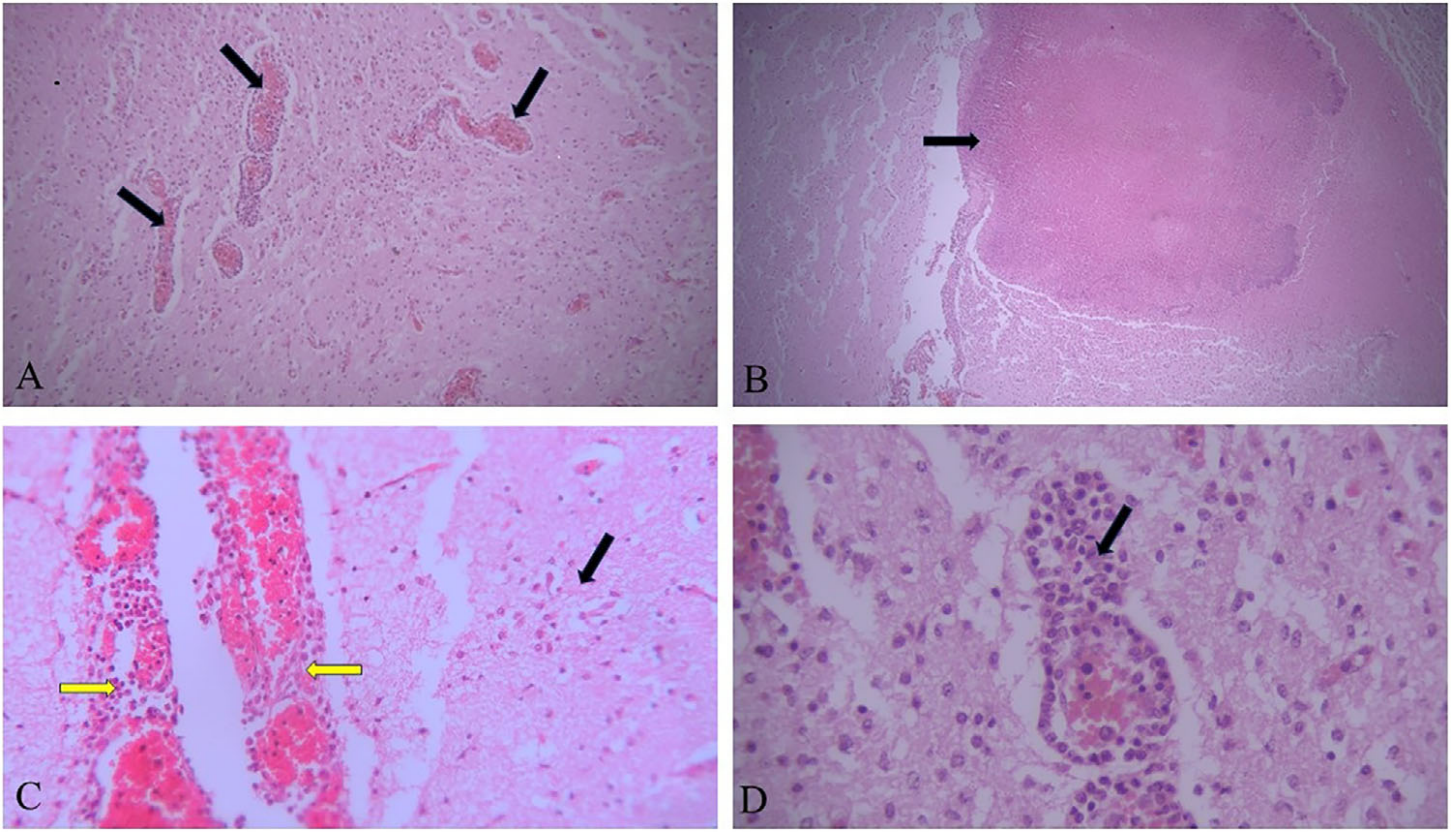
(A) Hyperemia and perivascular cuffing (H&E, ×10); (B) focal necrosis in foetal brain parenchyma (H&E, ×10); (C) hyperaemia and infiltration of inflammatory cells in meninges (yellow arrow) and astrogliosis (black arrow) (H&E, ×20); (D) severe perivascular cuffing (H&E, ×40).

**FIGURE 4:**
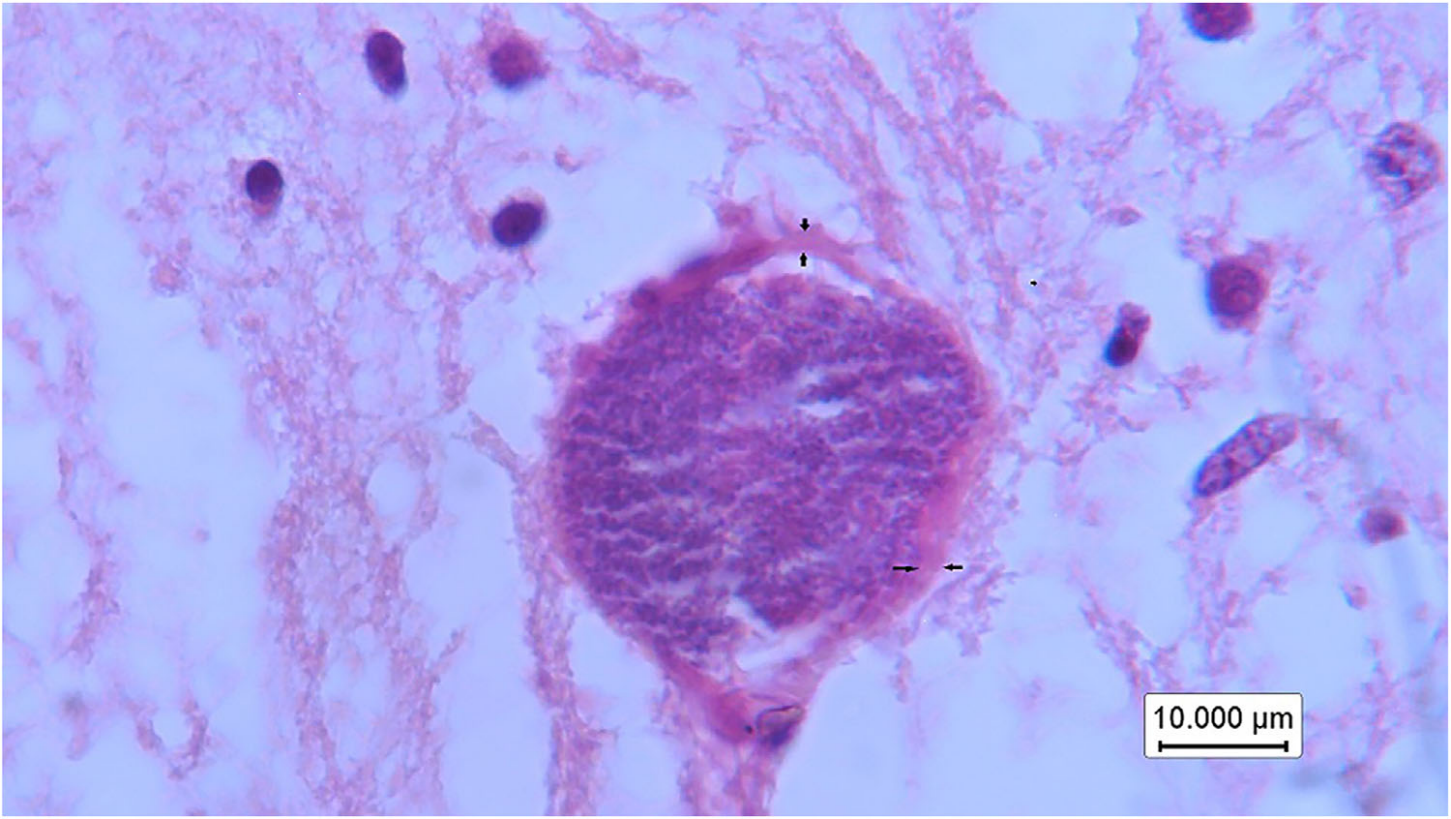
*Neospora caninum* cyst with thickness cell wall (black arrow) and numerous bradyzoites in the brain H&E, Barr = 10 µm (×100).

In this research, the gestational age of all bovine aborted foetuses was between 4 and 6 months, and 24.76% (26 out of 105) of the brain samples from these aborted foetuses were positive for *N. caninum* using nested PCR (Figure 2). Serum samples of all dams that aborted infected foetuses were positive for *N. caninum* antibodies by ELISA. In this study, many positive brain samples were autolysed after abortion, and only 20 brain tissue samples from 105 aborted foetuses were suitable for histopathological examination (Table 1). The pathology of the brain tissue revealed moderate‐to‐severe hyperaemia and perivascular cuffing in all cases (Figure 3). Several cysts measuring 25–44 µm in diameter, with a cyst walls thickness 2–2.5 µm was observed in one brain section of the 20 aborted foetuses. (Figure 4)

**TABLE 1:** Serological outcomes in the dam and histopathological results in their PCR‐positive aborted foetuses.

			Pathological findings in brain of aborted foetuses			
No.	Year and month of collection	Serology of dam	Hyperaemia	Perivascular cuffing	Necrosis	Cyst
1	02/2022	Positive	Positive	Positive	Negative	Negative
2	02/2022	Positive	Nd*	Nd	Nd	Nd
3	02/2022	Positive	Nd	Nd	Nd	Nd
4	03/2022	Positive	Nd	Nd	Nd	Nd
5	03/2022	Positive	Positive	Positive	Negative	Negative
6	03/2022	Positive	Positive	Positive	Positive	Positive
7	03/2022	Positive	Nd	Nd	Nd	Nd
8	04/2022	Positive	Positive	Positive	Negative	Negative
9	04/2022	Positive	Nd	Nd	Nd	Nd
10	04/ 2022	Positive	Positive	Positive	Negative	Negative
11	04/2022	Positive	Nd	Nd	Nd	Nd
12	04/ 2022	Positive	Positive	Positive	Negative	Negative
13	04/2022	Positive	Nd	Nd	Nd	Nd
14	05/ 2022	Positive	Nd	Nd	Nd	Nd
15	05/2022	Positive	Nd	Nd	Nd	Nd
16	02/ 2023	Positive	Nd	Nd	Nd	Nd
17	02/2023	Positive	Nd	Nd	Nd	Nd
18	02/2023	Positive	Positive	Positive	Negative	Negative
19	03/2023	Positive	Positive	Positive	Negative	Negative
20	03/2023	Positive	Positive	Positive	Negative	Negative
21	03/2023	Positive	Nd	Nd	Nd	Nd
22	03/2023	Positive	Positive	Positive	Negative	Negative
23	04/2023	Positive	Nd	Nd	Nd	Nd
24	04/2023	Positive	Positive	Positive	Negative	Negative
25	04/2023	Positive	Nd	Nd	Nd	Nd
26	05/2023	Positive	Positive	Positive	Negative	Negative

Abbreviation: Nd, not done due to post‐mortem changes.

## 4 Discussion

Neosporosis, caused by the parasite *N. caninum*, is a significant disease that affects livestock, particularly cattle, leading to abortions and substantial economic losses (Lefkaditis et al. 2020; Dubey et al. 2017; Maia et al. 2023). The current study utilized nested PCR and histopathological methods to detect *N. caninum* in aborted bovine foetuses. Among the diagnostic methods, PCR proved to be more sensitive and specific than other tests and was less influenced by autolysis and post‐mortem changes (Kamali et al. 2014). Our results showed that 24.76% of the sampled brains were PCR‐positive for *N. caninum*, which is consistent with the results of other studies in Iran and globally. For instance, studies in Iran have reported infection rates in aborted foetuses ranging from 11% to 75%, using various molecular techniques (Table 2). Similarly, international studies have reported varying prevalence rates: 50% in Australia (McInnes et al. 2006), 90% in Germany (Basso et al. 2010) and 90.6% in Argentina (Dorsch et al. 2021). The global prevalence of *N. caninum* infection in aborted bovine foetuses, as determined by PCR and nested PCR diagnostic methods, was estimated to be 41% and 50%, respectively (Table 3). Significant regional variations in *N. caninum* prevalence have been observed, which may arise from differences in diagnostic methodologies, study designs, sample types (such as blood, milk, semen and aborted materials), animal types and sample sizes used (Sykes et al. 2022).

**TABLE 2:** Characteristics of the included studies for the prevalence of *Neospora caninum* in aborted bovine foetuses by PCR in Iran.

Id	First author (Publication year)	Province	Sample	Methods	Sample size (*n*)	Molecular results *n* (%)
1	Habibi et al. (2005)	Khorasan	Brain	Semi‐nested PCR	6	4 (66.66)
2	Razmi et al. (2007)	Khorasan Razavi	Brain	Histopathology, IHC and PCR	100	13 (13)
3	Sadrebazzaz et al. (2007)	Khorasan	Foetal sera and fluids and brain	Histopathology, IFA and semi nested PCR	12	4 (33)
4	Salehi et al. (2009)	Tehran	Brain and placenta	Histopathology and nested PCR	19	17 (89.47)
5	Razmi et al. (2010)	Khorasan Razavi	Brain and foetal fluids	IHC, ELISA and PCR	151	18 (11.92)
6	Nematollahi et al. (2013)	East Azerbaijan	Brain, spinal cord, placenta, liver and heart	Histopathology and PCR	14	6 (42.86)
7	Razmi et al. (2013)	Khorasan Razavi	Brain	PCR	200	23 (11.5)
8	Kamali et al. (2014)	Iran	Brain	Histopathology and PCR	395	179 (45.31)
9	Salehi et al. (2015)	Tehran	Brain	Nested PCR	16	12 (75)
10	Kaveh et al. (2017)	Qazvin	Brain, kidney, spleen, liver and lung	PCR	128	39 (30.47)
11	Amouei et al. (2019)	Mazandaran	Brain	Nested PCR	9	2 (22.2)
12	Salehi et al. (2021)	Tehran	Brain	Nested PCR	78	16 (20.5)

**TABLE 3:** Characteristics of the included studies for the prevalence of *Neospora caninum* in bovine aborted foetuses by PCR in other countries.

Id	First author (Publication year)	Country	Sample	Methods	Sample size (*n*)	Molecular results *n* (%)
**1**	McInnes et al. (2006)	Australia	Foetal tissues	Nested PCR	42	21 (50)
**2**	Basso et al. (2010)	Germany	Brain	PCR	20	18 (90)
**3**	Medina‐Esparza et al. (2016)	Mexico	Brain	Nested PCR	63	27 (42.86)
**4**	Tian et al. (2018)	China	Foetal tissues	Nested PCR	75	17 (22.6)
**5**	Snak et al. (2018)	Brazil	Foetal tissues	PCR	17	9 (52.94)
**6**	Moroni et al. (2018)	Chile	Brain and optic nerve	PCR	296	31 (10.5)
**7**	Bartley et al. (2019)	Scotland	Brain, heart and placenta	Nested PCR	455	82 (18.02)
**8**	Serrano‐Martínez et al. (2019)	Peru	Foetal tissues	Nested PCR	68	11 (16.17)
**9**	Dorsch et al. (2021)	Argentina	Foetal tissues	Nested PCR	106	96 (90.6)
**10**	El‐Alfy et al. (2021)	Japan	Brain	Nested PCR	5	5 (100)
**11**	da Costa et al. (2022)	Brazil	Brain	PCR	28	20 (71.43)

Histopathological research has indicated that tissue cysts are predominantly localized in the central nervous system (Dubey et al. 2017). Therefore, the brain is considered the primary organ for diagnosing neosporosis in aborted foetuses (Dubey et al. 2017). Characteristic lesions of *N. caninum* infection, such as non‐suppurative encephalitis, were observed in 12 of 20 positive PCR brain samples, indicating a significant pathological impact of the parasite. These findings align with previous studies that have also noted non‐suppurative encephalitis, mononuclear cell infiltration, severe hyperaemia and perivascular cuffing as common lesions in *N. caninum*‐infected brains (Kamali et al. 2014; Salehi et al. 2009; Nematollahi et al. 2013; Moroni et al. 2018; Bartley et al. 2019). Additionally, histological examination of one brain sample revealed a few tissue cysts with thickness cyst walls that were similar to *N. caninum* cysts, the causative agent of neosporosis. This study, in agreement with some literature, used H&E staining to visualize *N. caninum* cysts within the brain tissue of aborted foetuses (Barr et al. 1990; Dubey et al. 2017; Morganti et al. 2024). *Neospora* tissue cysts can vary on the basis of the host species and strain of *Neospora*; studies generally report a range of approximately 5–50 µm in diameter (Dubey et al. 2017).

Serological analysis using ELISA revealed high levels of antibodies against *N. caninum* in all serum samples from cows with aborted foetuses, underscoring the importance of serological testing in diagnosing *N. caninum* infections. High seroprevalence rates have also been reported in other studies, reinforcing the reliability of serological tests as indicators of *N. caninum* exposure (Nayeri et al. 2022).

Regarding the advantages of serological testing, ELISA offers several benefits, including ease of use, cost‐effectiveness and the ability to quickly screen large numbers of samples. Additionally, serological tests can detect antibodies, even in the early stages of infection, providing valuable information for managing and controlling the spread of *N. caninum* in dairy herds. This is particularly important in regions with high prevalence rates, where early detection and intervention can mitigate the economic losses associated with bovine abortion (Wei et al. 2022).

## 5 Conclusion

Our findings support the significant role of *N. caninum* in causing abortions in dairy cattle, as evidenced by the molecular, histopathological, and serological data. High infection rates, consistent histopathological findings and reliable serological results highlight the importance of comprehensive diagnostic approaches for managing neosporosis. Preventive measures, such as preventing the consumption of placentas and aborted foetuses, should be considered to control the spread of *N. caninum* and to reduce its impact on the dairy industry. Although serology offers valuable information regarding exposure to *N. caninum*, a definitive diagnosis of *N. caninum*‐induced abortion hinges on a comprehensive foetal examination. This study, encompassing both histopathological analysis and potential PCR testing, provides the most reliable means of identifying the parasite and confirming its role in abortion.

## Author Contributions

**Asma Keyvanlou Shahrestanaki**: investigation, methodology, writing – review and editing, formal analysis. **Hossein Nourani**: methodology, writing – review and editing, writing – original draft. **Gholamreza Razmi**: supervision, writing – original draft, writing – review and editing, investigation, resources, formal analysis.

## Ethics Statement

The mice were housed and maintained in the animal care facility at Ferdowsi University of Mashhad. All animal experiments were performed in strict accordance with the guidelines approved by the Animal Ethics Committee of our faculty IR.UM.REC.1399.063.

## Conflicts of Interest

The authors declare no conflicts of interest.

### Peer Review

The peer review history for this article is available at https://publons.com/publon/10.1002/vms3.70329

## Data Availability

The datasets generated during and/or analysed during the current study are available from the corresponding author upon reasonable request.

## References

[vms370329-bib-0001] Abdelbaky, H. H. , M. Nishimura , N. Shimoda , et al. 2020. “Evaluation of *Neospora caninum* Serodiagnostic Antigens for Bovine Neosporosis.” Parasitology International 75: 102045. 10.1016/j.parint.2019.102045. 31881363

[vms370329-bib-0002] Amouei, A. , M. Sharif , S. Sarvi , et al. 2019. “Aetiology of Livestock Fetal Mortality in Mazandaran Province, Iran.” PeerJ 6: e5920. 10.7717/peerj.5920. 30687586 PMC6340351

[vms370329-bib-0003] Barr, B. C. , M. L. Anderson , P. C. Blanchard , B. M. Daft , H. Kinde , and P. A. Conrad . 1990. “Bovine Fetal Encephalitis and Myocarditis Associated With Protozoal Infections.” Veterinary Pathology 27: 354–361. 2238388 10.1177/030098589002700508

[vms370329-bib-0004] Bartley, P. M. , S. Guido , C. Mason , et al. 2019. “Detection of Neospora caninum DNA in Cases of Bovine and Ovine Abortion in the South‐West of Scotland.” Parasitology 146, no. 7: 979–982. 10.1017/S0031182019000301. 30975236

[vms370329-bib-0005] Basso, W. , S. Schares , L. Minke , et al. 2010. “Microsatellite Typing and Avidity Analysis Suggest a Common Source of Infection in Herds With Epidemic *Neospora caninum*—Associated Bovine Abortion.” Veterinary Parasitology 173, no. 1–2: 24–31. 10.1016/j.vetpar.2010.06.009. 20609521

[vms370329-bib-0006] Buxton, D. , S. Maley , S. Wright , K. Thomson , A. Rae , and E. Innes . 1998. “The Pathogenesis of Experimental Neosporosis in Pregnant Sheep.” Journal of Comparative Pathology 118, no. 4: 267–279. 10.1016/S0021-9975(07)80003-X. 9651804

[vms370329-bib-0007] da Costa, L. , J. A. Withoeft , J. V. Bilicki , et al. 2022. “ *Neospora caninum*—Associated Abortions in Cattle From Southern Brazil: Anatomopathological and Molecular Characterization.” Veterinary Parasitology: Regional Studies and Reports 36: 100802. 10.1016/j.vprsr.2022.100802. 36436886

[vms370329-bib-0008] de Souza, G. , L. Z. Amatti , L. V. Garcia , et al. 2022. “ *Neospora caninum* Infection and Reproductive Problems in Dairy Cows From Brazil: A Case‐Control Study.” Veterinary Parasitology: Regional Studies and Reports 28: 100683. 10.1016/j.vprsr.2021.100683. 35115122

[vms370329-bib-0009] Dorsch, M. A. , D. P. Moore , J. Regidor‐Cerrillo , et al. 2021. “Morphometric Study of Encephalic Lesions in Aborted Bovine Fetuses Naturally Infected by Two Subpopulations of *Neospora caninum* .” Parasitology Research 120, no. 8: 2995–3000. 10.1007/s00436-021-07248-y. 34292375

[vms370329-bib-0010] Dubey, J. P. , A. Hemphill , R. Calero‐Bernal , and G. Schares . 2017. Neosporosis in Animals. 1st ed. CRC Press. 10.1201/9781315152561.

[vms370329-bib-0013] El‐Alfy, E. , Y. Ohari , N. Shimoda , and Y. Nishikawa . 2021. “Genetic Characterization of *Neospora caninum* From Aborted Bovine Fetuses in Hokkaido, Japan.” Infection, Genetics and Evolution 92: 104838. 10.1016/j.meegid.2021.104838. 33819682

[vms370329-bib-0014] Gharekhani, J. , M. Yakhchali , and R. Berahmat . 2020. “ *Neospora caninum* Infection in Iran (2004–2020): A Review.” Journal of Parasitic Diseases 44, no. 4: 671–686. 10.1007/s12639-020-01266-w. 32929312 PMC7481549

[vms370329-bib-0015] Habibi, G. , R. Hashemi‐Fesharki , A. Sadrebazzaz , S. Bozorgi , and N. Bordbar . 2005. “Seminested PCR for Diagnosis of *Neospora caninum* Infection in Cattle.” Archives of Razi Institute 59, no. 2: 55–64. 10.22092/ari.2005.103813.

[vms370329-bib-0016] Idarraga‐Bedoya, S. , J. Álvarez‐Chica , D. K. Bonilla‐Aldana , D. P. Moore , and A. J. Rodríguez‐Morales . 2020. “Seroprevalence of *Neospora caninum* Infection in Cattle From Pereira, Colombia⋆.” Veterinary Parasitology: Regional Studies and Reports 22: 100469. 10.1016/j.vprsr.2020.100469. 33308726

[vms370329-bib-0017] Kamali, A. , H. A. Seifi , A. R. Movassaghi , G. R. Razmi , and Z. Naseri . 2014. “Histopathological and Molecular Study of *Neospora caninum* Infection in Bovine Aborted Fetuses.” Asian Pacific Journal of Tropical Biomedicine 4, no. 12: 990–994. 10.12980/APJTB.4.201414B378.

[vms370329-bib-0018] Kaveh, A. , E. Merat , S. Samani , S. Danandeh , and S Soltan Nezhad . 2017. “Infectious Causes of Bovine Abortion in Qazvin Province, Iran.” Archives of Razi Institute 72, no. 4: 225–230. 10.22092/ari.2017.113299. 30315698

[vms370329-bib-0019] Khan, A. , J. S. Shaik , P. Sikorski , J. P. Dubey , and M. E. Grigg . 2020. “Neosporosis: An Overview of Its Molecular Epidemiology and Pathogenesis.” Engineering 6, no. 1: 10–19. 10.1016/j.eng.2019.02.010.

[vms370329-bib-0020] Lefkaditis, M. , R. Mpairamoglou , A. Sossidou , K. Spanoudis , and M. Tsakiroglou . 2020. “ *Neospora caninum*, a Potential Cause of Reproductive Failure in Dairy Cows From Northern Greece.” Veterinary Parasitology: Regional Studies and Reports 19: 100365. 10.1016/j.vprsr.2019.100365. 32057391 PMC7103951

[vms370329-bib-0021] Maia, A. , R. P. B. de Melo , R. A. Mota , et al. 2023. “Herd and Animal Level Prevalences and Risk Factors for *Neospora caninum* Infection in Cattle in the State of Paraíba, Northeastern Brazil.” Veterinary Parasitology: Regional Studies and Reports 40: 100866. 10.1016/j.vprsr.2023.100866. 37068861

[vms370329-bib-0022] McInnes, L. M. , U. M. Ryan , R. O'Handley , H. Sager , D. Forshaw , and D. G. Palmer . 2006. “Diagnostic Significance of *Neospora caninum* DNA Detected by PCR in Cattle Serum.” Veterinary Parasitology 142, no. 3–4: 207–213. 10.1016/j.vetpar.2006.07.013. 16934934

[vms370329-bib-0042] McAllister, M. M. , J. P. Dubey , D. S. Lindsay , W. R. Jolley , R. A. Wills , and A. M. MacGuire . 1998. “Dogs are definitive hosts of *Neospora caninum* .” International Journal of Parasitology 28, no. 9: 1473–1478. 9770635

[vms370329-bib-0023] Medina‐Esparza, L. , J. Regidor‐Cerrillo , D. García‐Ramos , et al. 2016. “Genetic Characterization of *Neospora caninum* From Aborted Bovine Foetuses in Aguascalientes Mexico.” Veterinary Parasitology 228: 183–187. 10.1016/j.vetpar.2016.09.009. 27692324

[vms370329-bib-0024] Morganti, G. , G. Rigamonti , L. Brustenga , et al. 2024. “Exploring Similarities and Differences Between *Toxoplasma gondii* and *Neospora caninum* Infections in Dogs.” Veterinary Research Communications 48, no. 6: 3563–3577. 10.1007/s11259-024-10549-z. 39320405 PMC11538173

[vms370329-bib-0025] Moroni, M. , M. Navarro , E. Paredes , et al. 2018. “Identification of *Neospora caninum* in Aborted Bovine Fetuses of Southern Chile.” Brazilian Journal of Veterinary Pathology 11, no. 2: 37–41. 10.24070/bjvp.1983-0246.v11i2p37-41.

[vms370329-bib-0026] Nayeri, T. , M. Moosazadeh , S. Sarvi , and A. Daryani . 2022. “ *Neospora caninum* Infection in Aborting Bovines and Lost Fetuses: A Systematic Review and Meta‐Analysis.” PLoS ONE 17, no. 5: e0268903. 35604902 10.1371/journal.pone.0268903 PMC9126370

[vms370329-bib-0027] Nematollahi, A. , G. Moghaddam , R. Jaafari , J. A. Helan , and M. Norouzi . 2013. “Study on Outbreak of *Neospora caninum*—Associated Abortion in Dairy Cows in Tabriz (Northwest Iran) by Serological, Molecular and Histopathologic Methods.” Asian Pacific Journal of Tropical Medicine 6, no. 12: 942–946. 10.1016/S1995-7645(13)60168-6. 24144024

[vms370329-bib-0028] Otranto, D. , A. Llazari , G. Testini , et al. 2003. “Seroprevalence and Associated Risk Factors of *Neosporosis* in Beef and Dairy Cattle in Italy.” Veterinary Parasitology 118, no. 1–2: 7–18. 10.1016/j.vetpar.2003.10.008. 14651870

[vms370329-bib-0029] Razmi, G. , H. Zarae , M. F. Norbakhsh , and Z. Naseri . 2013. “Estimating the Rate of Transplacental Transmission of *Neospora caninum* to Aborted Fetuses in Seropositive Dams in Mashhad Area, Iran.” Iranian Journal of Veterinary Medicine 7, no. 4: 253–256. 10.22059/ijvm.2013.36284.

[vms370329-bib-0030] Razmi, G. , H. Zarea , and Z. Naseri . 2010. “A Survey of *Neospora caninum*—Associated Bovine Abortion in Large Dairy Farms of Mashhad, Iran.” Parasitology Research 106: 1419–1423. 10.1007/s00436-010-1820-3. 20352453

[vms370329-bib-0031] Razmi, G. R. , M. Maleki , N. Farzaneh , M. Talebkhan Garoussi , and A. H. Fallah . 2007. “First Report of *Neospora caninum*—Associated Bovine Abortion in Mashhad Area, Iran.” Parasitology Research 100: 755–757. 10.1007/s00436-006-0325-6. 17024355

[vms370329-bib-0032] Sadrebazzaz, A. , G. Habibi , H. Haddadzadeh , and J. Ashrafi . 2007. “Evaluation of Bovine Abortion Associated With *Neospora caninum* by Different Diagnostic Techniques in Mashhad, Iran.” Parasitology Research 100: 1257–1260. 10.1007/s00436-006-0417-3. 17206503

[vms370329-bib-0033] Salehi, B. , A. Amouei , S. Dodangeh , et al. 2021. “Molecular Identification of *Neospora caninum* Infection in Aborted Fetuses of Sheep, Cattle, and Goats in Mazandaran Province, Northern Iran.” Iranian Journal of Parasitology 16, no. 3: 483–489. 34630594 10.18502/ijpa.v16i3.7102 PMC8476731

[vms370329-bib-0034] Salehi, N. , B. Gottstein , H. Haddadzadeh , J. Ashrafihelan , P. Shayan , and A. Sadrebazzaz . 2009. “Molecular and Pathological Study of Bovine Aborted Fetuses and Placenta From *Neospora caninum* Infected Dairy Cattle.” Iranian Journal of Parasitology 4: 40–51.

[vms370329-bib-0035] Salehi, N. , B. Gottstein , and H. Haddadzadeh . 2015. “Genetic Diversity of Bovine *Neospora caninum* Determined by Microsatellite Markers.” Parasitology International 64, no. 5: 357–361. 10.1016/j.parint.2015.05.005. 25988829

[vms370329-bib-0036] Serrano‐Martínez, M. E. , C. A. B. Cisterna , R. C. E. Romero , M. A. Q. Huacho , A. M. Bermabé , and L. A. L. Albornoz . 2019. “Evaluation of Abortions Spontaneously Induced by *Neospora caninum* and Risk Factors in Dairy Cattle From Lima, Peru.” Revista Brasileira De Parasitologia Veterinária 28: 215–220. 10.1590/S1984-29612019026. 31215607

[vms370329-bib-0037] Snak, A. , F. G. Garcia , A. A. Lara , H. F. J. Pena , and S. C. Osaki . 2018. “ *Neospora caninum* in Properties in the West Region of Paraná, Brazil: Prevalence and Risk Factors.” Revista Brasileira De Parasitologia Veterinária 27: 51–59. 10.1590/S1984-29612018001. 29641794

[vms370329-bib-0038] Sykes, J. , M. Lappin , and J. Dubey . 2022. Neosporosis, in Greene's Infectious Diseases of the Dog and Cat. Edited by J. E. Sykes . Elsevier: USA. 10.1016/B978-0-323-50934-3.00094-X.

[vms370329-bib-0039] Tian, A. , H. M. Elsheikha , D. Zhou , et al. 2018. “A Novel Recombinase Polymerase Amplification (RPA) Assay for the Rapid Isothermal Detection of *Neospora caninum* in Aborted Bovine Fetuses.” Veterinary Parasitology 258: 24–29. 10.1016/j.vetpar.2018.06.004. 30105974

[vms370329-bib-0040] Wei, X. , Q. An , N. Xue , et al. 2022. “Seroprevalence and Risk Factors of *Neospora caninum* Infection in Cattle in China From 2011 to 2020: A Systematic Review and Meta‐Analysis.” Preventive Veterinary Medicine 203: 105620. 10.1016/j.prevetmed.2022.105620. 35366534

[vms370329-bib-0041] Zaghawa, A. , S. Elgendy , M. Nayel , et al. 2023. “Neosporosis in Farm Animals.” Journal of Current Veterinary Research 5, no. 1: 87–109. 10.21608/jcvr.2023.296041.

